# Facts about Families

**Published:** 1895-03-23

**Authors:** 


### Modern Sociology.

### FACTS ABOUT FAMILIES.

One of the most popular subjects for letters to the newspapers (written mostly in the editorial office) during the dull season is the question of marriage,—why men don't marry and why they do, whether women who marry repent or not, and so forth. If the writers would only consult the statistics of the subject it would save them a world of conjecture, but it would stop the correspondence! In spite of newspaper pessimists, men marry when they can afford it. The marriage rate of a country depends, in the first place, on the number of marriageable men it numbers, and in the second on the prosperity it attains. Other things, indeed, modify these rules. For example, men marry more readily in an agricultural community, where a wife is a helper, than in a city where she is merely a luxury; and in new colonies men remain unmarried for lack of suitable wives, while at home their female relatives pine and—dare we suggest it in these latter days?—cry "Heigh-ho for a husband." If it seems to us in Britain that there are fewer marriages now than of yore, the reason is that our adventurous sons go to Central Africa while our adventurous daughters go no farther than London, and so are still numbered by the Registrar-General. But if our spinsters largely out-number our bachelors, it must be remembered that although more boys than girls are born, fewer attain to adult life. About 120 male children die for 100 female. The average proportion of male to female births is about 105 to 100, but it must be said that in England the proportion is not so high. The average here is 104·3 and the Registrar General declares that during the last fifty years the proportions of the sexes have shown a tendency to approximate. No explanation of the fact is offered, but perhaps some guidance might be gained from studying the conditions of life in Greece, which is emphatically the mother of men. The birth-rate there averages 112 boys to 100 girls. It can hardly be a matter of race alone, for in Connecticut, which is almost as English as our own country, the proportion is 110 to 100. But in the Australian colonies the numbers are but little higher than our own. Perhaps it is because our margin of men is so small that the "woman question" is so acute with us.

England is known to foreign observers as the country of large families. "The Queen," says even the Anglophil Taine, "sets the fashion." Yet it is not England itself that has the highest average. Ireland, the distressful country, which has a marriage rate of only 4·4 per thousand of population, heads the list with an average of 5·46 children to each marriage. If it were not for this high birth-rate, the population of Ireland would probably, in view of the large and constant stream of emigration from it, decrease rapidly. Next in order come all the Australasian colonies, with the exception of Victoria, ranging from New Zealand, with 5·21 children to each marriage, to Queensland with 4·60. Victoria comes modestly after Italy, Scotland, and Holland with 4·20. Scotland shows an average of 4·43, while England does not exceed 4·16. After England come Sweden, Denmark, and France. The last averages only 2·98 children in a family. Thus the largest possible increase in population in France would be small, and allowing for the deaths of children we find that it is almost stationary This, as is well known, is the result of the prudence of the Frenchman, who, having worked to attain a modest competence, does not like the idea of it being divided among a too numerous progeny. He would rather have a small family whom he can provide for adequately. The same idea prevails, though less consciously in all civilized countries. A greater amount of luxury in domestic customs tends inevitably to postpone marriage until a later period in a man's life. He cannot while young keep a wife according to the standard of comfort of his class, and delays until he approaches middle age. Sometimes at a mature age he marries a young girl, but not so often as the novelists would make us believe. The ages of both brides and bridegrooms are greater now than they were a generation or two back. In his grave Malthus has conquered. Marriage is postponed, and families are smaller in consequence.

We have spoken of the large size of Irish families. Considering the poverty of the nation it might be thought strange that, not only are many children born, but that so large a proportion of them are successfully reared. Ireland shows almost the lowest record of infantile mortality, New Zealand alone surpassing her. The first year of life is that in which the dangers are greatest, but statistics show that in Ireland barely 10 per cent. of the children die before completing their first year. New Zealand, as we have said, has a better record even than this, the proportion there being 8·74 deaths to every hundred births. England's infant death-rate shows a proportion of 14·92 to the hundred, and Scotland stands midway with 12·20. When one considers the lack of the first essentials of comfort in an Irish peasant's hut, it seems strange that they, of all people, should be so successful in bringing their children through the dangers of infancy, but perhaps, the explanation lies in their being *paysans*. It is the great towns with their smoke-dimmed air that slay the children; a pure atmosphere may atone for the lack of many luxuries. The German Empire and Austria, however, cut the worst figure in these statistics of infant mortality. The death-rate there varies from 31·25 per cent. in Wurtemburg to 20·78 in Prussia. Italy is a little worse than Prussia, with 20·79, while Holland is rather better, her ratio standing at 19·32. It is curious that Sweden and Norway, closely as they are connected in every way, should show such different records in this matter. In Sweden the deaths are comparatively numerous, 13·19 to 100 births; in Norway they are only 10·49. Between the figures of the two Scandinavian countries come those of the Australian colonies, Victoria heading the list with 12·68, and Tasmania ending it with 10·56.

Taking the general death-rate, however, the same proportions are not maintained. Reckoning by the excess of births over deaths at all ages, we find Norway and Sweden together at the head of the list with 78 and 75 respectively. England and Scotland stand even closer, the numbers in them being 64 and 63. Ireland comes low down with an excess of only 27. But when one recalls how high the absolute birth-rate is, one can only assume that the green isle is so healthy that people can't die there even if they want to.

